# *In silico* evaluation of missense SNPs in cancer-associated Cystatin A protein and their potential to disrupt Cathepsin B interaction

**DOI:** 10.1016/j.heliyon.2025.e42478

**Published:** 2025-02-05

**Authors:** Shafaat Hossain, Omar Hamza Bin Manjur, Mst Sharmin Sultana Shimu, Tamanna Sultana, Mustafizur Rahman Naim, Shahariar Siddique, Abdullah Al Mamun, Md Miftaur Rahman, Md Abu Saleh, Md Rakibul Hasan, Tania Rahman

**Affiliations:** a Department of Biology & Biochemistry, University of Houston, USA; b Department of Biochemistry & Molecular Biology, University of Dhaka, Bangladesh; c Bangladesh Reference Institute for Chemical Measurements (BRiCM), Bangladesh; d Department of Genetic Engineering and Biotechnology, University of Rajshahi, Rajshahi, Bangladesh; e Biomedical and Toxicological Research Institute (BTRI), Bangladesh Council of Scientific and Industrial Research (BCSIR), Dhaka, Bangladesh; f Institute of Food Science and Technology (IFST), Bangladesh Council of Scientific and Industrial Research (BCSIR), Dhaka, Bangladesh; g Department of Biochemistry & Biotechnology, University of Science and Technology, Chittagong, Bangladesh; h Institute of Technology Transfer and Innovation (ITTI), Bangladesh Council of Scientific and Industrial Research (BCSIR), Dhaka, Bangladesh; i Bangladesh Dental College, Dhaka, Bangladesh

**Keywords:** Stefin A, Docking, Molecular dynamics, Homology modeling, Metastasis, COSMIC

## Abstract

Cystatin A (CSTA) functions as a cysteine protease inhibitor by forming tight complexes with the cathepsins. Pathogenic mutations in the CSTA gene can disrupt this interaction, potentially leading to physiological ailments. In this study, eight bioinformatics tools (SIFT, PolyPhen-2, PROVEAN, P-Mut, MutPred2, SNAP2, SNPs & GO, and PHD-SNP) were implemented to analyze non-synonymous SNPs from the dbSNP database. Five mutations (Y43C, Y43N, V48F, Y53H, and E94K) located in the conserved region were found to be highly deleterious and less stabilizing. The protein-protein interaction network found that Cathepsin B (CTSB) interacts highly with CSTA. Mutated CSTAs were created by homology modeling, and their altered binding with CTSB was examined through molecular docking and dynamics simulations. Among these, the Y53H (rs1448459675) and E94K (rs200394711) mutants were recognized as weaker inhibitors because they had 2.5 % and an 8 % lower binding affinity, respectively. Moreover, the E94K-CTSB complex, with a root mean square deviation (RMSD) above 5 Å, was found to be highly unstable during molecular dynamics. The root mean square fluctuation (RMSF) of the E94K mutant showed insufficient flexibility, indicating a reduced capacity to suppress CTSB. These findings suggest that the E94K mutation could affect the protein structure and cathepsin B interaction, potentially leading to pathological consequences as evidenced by colorectal adenocarcinoma patients in the COSMIC (Catalogue of Somatic Mutations in Cancer) database.

## Highlights

• Five nsSNPs were detected as deleterious, destabilizing and evolutionarily conserved.
• CTSB binding affinity is decreased by the Y53H-CSTA and E94K-CSTA mutants.
• MD simulation showed high RMSD for E94K-CTSB complex resembled weak inhibition.
• E94K mutation is found in adenocarcinoma patients, indicating clinical significance.

## 1 Introduction

A single nucleotide polymorphism (SNP) is described as a variation of a single nucleotide at a particular position within the genome, with each variation existing to some perceptible degree within a population. SNPs are reported to be frequent and are considered to take place at 1 out of every 1000 bases in the human genome [1], making up around 90 % of all genetic variations [2]. If an SNP occurs at the coding region of a gene and consequently alters the structure or function of the encoded protein, it is known as a missense or nsSNP. Close to half of the genetic variations linked to inherited human diseases can be attributed to nsSNPs, and they are therefore routinely analyzed for diagnostic purposes [3]. Missense SNPs are reported to be strongly affiliated with a wide range of disease phenotypes, which include attention deficit hyperactivity disorder [4], bipolar affective disorder, major depressive disorder, schizophrenia [5], type 1 diabetes, autoimmune diseases [6], and breast and ovarian cancer [7], among others.

Cystatins constitute a large superfamily functioning as reversible competitive inhibitors of C1 cysteine proteases [8]. While initially the primary role of the cystatins was thought to be as regulators of cysteine protease activity, later this superfamily was found to be associated with a multitude of other cellular and physiological functions such as immunomodulation, cell survival, signaling, proliferation, and differentiation [9,10]. In higher eukaryotes, the cystatin superfamily is comprised of five distinct families: stefin, cystatin, kininogen, latexin, and fetuin; among them, the first three in the list are considered to be the major families [8,9]. X-ray crystallography and mutagenesis exhibited three conserved regions in cystatins: an N-terminal glycine region, a glutamine-valine-glycine (Q-X-V-X-G) conserved loop, and a second C-terminus hairpin loop. The wedge-shaped structure formed by these motifs blocks the active site of C1 cysteine [11,12]. Functional aberrations in cystatins have been implicated in the progression of a wide array of pathological conditions, such as arthritis, atherosclerosis, amyloidosis, and cancer [10].

Cystatin A (CSTA) belongs to the Stefin family and functions as a suppressor of cysteine proteases, especially cathepsins B, H, and L [13]. It is essential for maintaining epithelial integrity by regulating enzymes to prevent uncontrolled proteolysis, leading to tissue destruction, inflammation, or disease progression [14]. Keratinocytes within the skin highly express CSTA protein by preserving the structural and functional integrity of the epidermal barrier [15]. The function is crucial for preventing and managing skin disorders like psoriasis, eczema, and atopic dermatitis because increased protease activity disrupts skin defense mechanisms [16,17]. The presence of high CSTA levels in tumor tissues or patient blood has been observed in a number of malignancies, including breast [18], colorectal [19], head and neck [20], lung [[21], [22], [23]], and hepatocellular carcinoma [24,25]. These increased levels are commonly associated with advanced disease and a poor prognosis [26]. High extracellular CSTA levels in colorectal carcinoma are linked to shorter survival time, indicating their involvement in tumor growth [27]. Similarly, nasopharyngeal carcinoma (NPC) patients with elevated levels of CSTA in their blood have poor prognostic outcomes [26]. On the other hand, in other malignancies, such as prostate cancer, elevated CSTA levels are connected to less severe disease, which highlights the dual nature of the role that CSTA plays in cancer [28]. Conversely, in cancers like oral squamous cell carcinoma (OSCC), esophageal squamous cell carcinoma (ESCC), and prostate cancer, decreased CSTA expression is frequently observed and strongly associated with aggressive tumor phenotypes, as well as high tumor grades, nodal metastasis, and poor overall survival [29,30]. Mechanistic findings indicate that the overexpression of CSTA in squamous cell carcinoma (OSCC) inhibits epithelial-to-mesenchymal transition (EMT), hence reducing the migration and invasion of tumor cells [31]. The suppression of the ERK/MAPK signaling pathway and the modulation of tumor-stroma interactions are mechanisms responsible for this effect [32]. Furthermore, CSTA is recognized for its ability to inhibit cathepsin B (CTSB), a cysteine protease implicated in the degradation of the extracellular matrix and tumor growth. In case of the breast [33], brain [34], and prostate malignancies [35], the lack of CSTA results in unregulated CTSB activity, facilitating invasive and metastatic behavior in tumors. In the case of esophageal squamous cell carcinoma (ESCC), for instance, early tumors display a much lower level of CSTA expression, but more advanced tumor regions display relatively higher levels, which may be a reaction to increased protease activity [36]. The relationship between CSTA and cathepsins illustrates the balance between the two, with disruptions in their interaction playing a role in cancer progression [32]. The molecular mechanisms through which CSTA exerts tumor-suppressing or oncogenic effects remain poorly understood, even though most studies have emphasized the correlation between CSTA expression levels and clinical outcomes only.

The specific function of CSTA in cancer progression, particularly the impact of different amino acid alterations on cystatin-cathepsin interactions, is still not fully elucidated. In this study, the most significant nsSNPs of CSTA have been shortlisted and further explored by their altered structural features through *in silico* tools. This analysis will provide a framework for future *in vitro* and case-control studies to describe the structural and functional impact of the SNPs in the CSTA gene and observe the cystatin-cathepsin interaction pattern.

## 2 Methods

### 2.1 SNP mining

SNPs associated with the human CSTA gene were retrieved from the Ensembl genome browser, selecting dbSNP as the source database [37]. Only the missense variants were filtered out. The representative protein sequence has been collected from UniProt [38] (UniProt ID: P01040) for further bioinformatics analysis.

### 2.2 Identifying the most deleterious nsSNPs

The functional consequences of nsSNPs were evaluated by employing eight computational tools: SIFT, PolyPhen-2, PROVEAN, P-Mut, MutPred2, SNAP2, SNPs & GO, and PHD-SNP. SIFT performs a sequence-homology dependent approach to assess the deleterious outcome of nsSNPs. SNPs with a sift value less than 0.05 are anticipated to be deleterious [39]. PolyPhen-2 calculates the probable effect of amino acid substitutions on protein structure and functionality. It analyzes an independent position-specific count score for every SNP fluctuating from 0 to 1, where 1 is considered most detrimental, and 0 indicates benign effect [40]. PROVEAN tool predicts whether a substituted amino acid or indel has a functional impact on protein. It determines a score for every variant from the homologous sequence alignment. Variants with a score ≤ −2.5 are assumed to be deleterious [41]. PMut permits a rapid neural network-based assumption of the pathological character in each point mutation in CSTA. A score above 0.5 indicates that nsSNPs have a detrimental influence on that protein function [42]. MutPred2 analyzes the outcome of nsSNPs on tertiary protein structure by receiving the FASTA sequence, the site of the mutation, and the wild and mutant types. The results include a MutPred2 score (>0.50 is measured as pathogenic), modified PROSITE and ELM motifs, and a table containing the structural dissimilarities that occurred, such as the loss or gain of an alpha helix, beta strand, or loop [43]. Based on a neural network classification algorithm, SNAP2 predicts the functional effects of amino acid variation. This server takes FASTA sequences and indicates the chance that a particular mutation would change the function of the original protein [44,45]. PhD-SNP uses the support vector machine (SVM) algorithm to determine if an nsSNP phenotype may be linked to any disease-associated circumstances. A reliability index that indicates whether the SNP is disease-causing or neutral is included in the output [46,47]. Another SVM-based technique, SNPs & GO, predicts whether a mutation will result in illness based on the protein sequence, protein structure (if available), and gene ontology (GO) terms [48,49]. The nsSNPs in CSTA protein predicted to be deleterious and disease-associated by all eight *in silico* tools were classified as high-risk and subjected to further downstream investigations.

### 2.3 Evaluation of changes in protein stability through nsSNP analysis

The free energy associated with unfolding a protein is a crucial measure of its stability. Protein stability tests were subsequently performed on the high-risk nsSNPs of CSTA using the I-Mutant 2.0, MUpro, and INPS tools. I-Mutant 2.0 predicts changes in protein stability automatically in response to single-site mutations [50]. Analyzing the mutated protein structure or sequence makes it possible to determine the magnitude and direction of the alteration in free energy [46,47]. All submissions were maintained at physiological temperature (37 °C) and pH (7.0). MUpro is an assembly of machine learning algorithms established to forecast how a single-site amino acid change may impact the stability of proteins [51], which gives a score between −1 and 1 to estimate the confidence of the prediction. A score less than zero indicates that the mutation has a negative impact on the stability of the protein. On the other hand, a score greater than zero indicates that the mutation enhances the stability of the protein [51]. INPS determines the affected stability of proteins by their nsSNPs. By calculating the energy level of protein with or without variation, the server computes the change in thermodynamic stability (DDG score) [52]. nsSNPs with decreasing protein stability in all three tools were selected.

### 2.4 Conservancy analysis

The evolutionary conservation of an amino acid position indicates its structural and functional significance [53]. ConSurf server was used to access amino acid substitutions in evolutionarily conserved regions, determining the evolutionary rate using Bayesian or maximum likelihood (ML) methods [54,55]. The FASTA sequence of CSTA protein was used to calculate scores and color schemes, identifying high-risk nsSNPs and their significant conservation. Additionally, it utilizes the NACSES algorithm to determine if a specific amino acid residue is exposed or buried [53].

### 2.5 Prediction of structural changes due to nsSNPs

Project HOPE (Have (y) Our Protein Explained) webserver was used to forecast how changes in amino acid composition would affect the CSTA protein's structural makeup. This server uses the proteins' tertiary structures from the Distributed Annotation System and UniProt database to evaluate the structural implications of mutations on the native protein [56]. Project HOPE accepts protein sequences and consecutive point mutations as inputs. The results are obtained by finding structural differences between mutant and wild-type residues and analyzing the overall impact of amino acid alterations.

### 2.6 Protein-protein interaction analysis

Analysis of protein-protein interactions is crucial for comprehending intracellular interactions, the impact of defective proteins on the network, and their connection to illness. The STRING database was utilized to identify the proteins that closely interact with CSTA. Parameters used for these predictions included gene fusions, co-occurrence, co-expression, as well as experimental and biochemical data. The combined score for each protein's interaction with the target protein ranged from 0 to 1, with 0 representing the weakest interaction and 1 representing the strongest [57,58]. The network was generated to identify the interacting protein partners with a high confidence value of 0.700 [59]. Finally, the partner protein with the highest score was chosen for downstream molecular docking and dynamics studies.

### 2.7 Homology modeling, structure optimizing, and visualization

The 3D structure of the wild-type CSTA protein (PDB ID: 3KSE) as a ligand and the CTSB protein (PDB ID: 3K9M) as the receptor was obtained from the RCSB-PDB. These proteins were processed by eliminating all heteroatoms and water molecules using the PyMOL software package (version 1.3) [60]. In addition, the three-dimensional structures of mutant CSTAs were generated by homology modeling using SWISS-MODEL, with wild-type protein as a template. This process involves template identification, sequence alignment, generating several models, and finally, model quality assessment based on the QMEAN, GMQE, and QMEANDisCo Score [61]. The GalaxyRefine tool was employed to refine models and enhance Rama-favored regions. It is a web server that specializes in the refinement of protein structures, with a particular emphasis on increasing the quality of local structures to refine loop or terminal sections [62]. Finally, MolProbity was used to verify all the mutant structures produced by homology modeling. A Ramachandran plot was constructed to validate the structure by using data on residues in the core and other permissible positions [63]. Discovery Studio 4.169 was utilized to visualize 3D constructions of both wild-type and mutant CSTAs and the intramolecular changes associated with those mutations [64].

### 2.8 Protein-protein docking

Protein-protein docking predicts interactions by utilizing steric and physicochemical complementarity at the protein-protein interface. The CSTA proteins, whether normal or mutant, and CTSB were submitted as ligand and receptor molecules, respectively, to the HDOCK server. This server implements a rapid Fourier transform search method that integrates template-based modeling and *ab initio* free docking in a hybrid framework [55,56]. The HDOCK server generates a docking score based on either the ITScorePP or ITScorePR. A lower docking score signifies a higher probability of a binding model [65]. The CSTA-CTSB complexes that were docked and had the lowest docking energy scores were chosen as the most accurate binding model. The results were confirmed by submitting them to the PRODIGY webserver. This tool was utilized to evaluate the binding strength, dissociation constant, and quantity of interactions formed between protein complexes at 37 °C. Complexes exhibiting the lowest energy value (ΔΔG) possess a higher binding affinity and correlate with mutations that enhance the stability of protein structures [66,67]. After that, the binding complexes of target proteins were obtained by PyMoL and their interactions are visualized in Discovery Studio 4.1.

### 2.9 Molecular dynamics simulation

The docked CSTA-CTSB complexes were simulated using the GROningen MAchine for Chemical Simulations, or GROMACS (version 2020.6) [68]. The force field employed for the simulation was CHARMM36m. A water box with edges that are 1 nm away from the protein surface was constructed using the TIP3 water model. Appropriate ions had been utilized to neutralize the simulation environment. A 100 ns molecular dynamic simulation was performed utilizing periodic boundary conditions and a temporal integration step of 2 fs after energy minimization, isothermal-isochoric, and isobaric system equilibrium. To assess the trajectory data, the snapshot interval was set to 100 ps. After the simulation was complete, the root mean square deviation (RMSD), root mean square fluctuation (RMSF), radius of gyration (Rg), and solvent accessible surface area (SASA) analyses were performed using the “rms”, “rmsf”, “gyrate”, and “sasa” modules integrated into the GROMACS program. The plots for this study were produced using the ggplot2 package in RStudio. The high-performance simulation stations employing the Ubuntu 20.04.4 LTS operating system were used for all molecular dynamics simulations.

## 3 Results

**Fig. 1:**
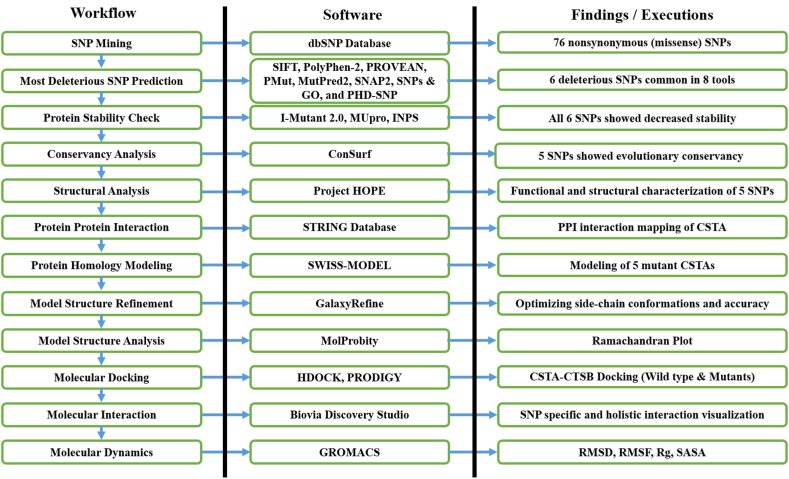
A flowchart illustrating the complete workflow, software used, and outcomes obtained from this study.

Fig. 1 illustrates the comprehensive study design, encompassing all the various software employed and their corresponding outcomes.

### 3.1 CSTA gene variants identifies 76 non-synonymous SNPs

**Fig. 2:**
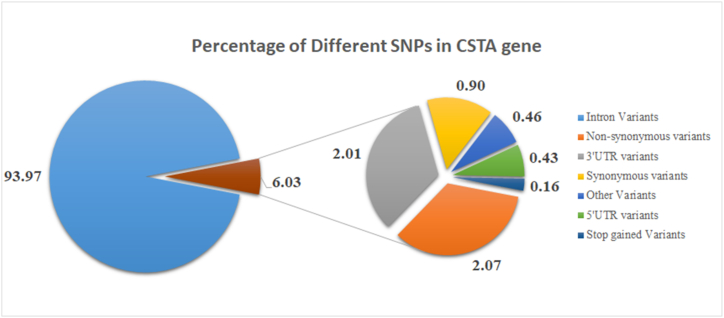
The left pie chart shows the proportion of intronic variations compared to the total number of variants. The right pie chart illustrates the breakdown of additional SNPs from the first pie chart.

The nsSNPs of the CSTA gene thoroughly scrutinized in this study were retrieved from the NCBI dbSNP database with transcript ID ENST00000264474.4. Based on their rsIDs, a total of 3680 SNPs for the CSTA gene were found. Of these, 76 were non-synonymous (missense) variants, 33 were synonymous variants, 3458 were intronic variants, 74 were in the 3′UTR region, 16 were in the 5′UTR region, 6 were stop-gained variants, and the rest were other types (Fig. 2). Only nsSNPs were considered for downstream analysis since deleterious nsSNPs could potentially cause structural and functional changes in the protein [69].

### 3.2 Eight multi-categorical computational tools identifies six potentially deleterious nsSNPs

A diverse range of computational tools is available for estimating the impact of missense SNPs on protein function. These tools were categorized into three classes: sequence-based, structural and functional prediction-based, and disease and functional annotation-based. The SIFT and PolyPhen-2 tools, part of the sequence-based category, were initially used to narrow down the number of nsSNPs for further analysis. Only nsSNPs with a SIFT score <0.05 and a PolyPhen score >0.90 were selected. This stringent selection process led to the identification of 14 out of 76 nsSNPs as deleterious by both tools (Table 1, supplementary file 1). After that, tools from the remaining two categories were used: disease and functional annotation (P-Mut, PHD-SNP, and SNPs & GO) and structural and functional prediction (PROVEAN, MutPred2, and SNAP2) (Table 2). Six nsSNPs (rs1262778803 (Y43C), rs1486315855 (Y43N), rs1559982616 (V47F), rs745733445 (V48F), rs1448459675 (Y53H), rs200394711 (E94K)) were found to be deleterious by all eight computational tools used. These high-risk nsSNPs were chosen for further analysis.

**Table-1:** Deleterious nsSNPs predicted by SIFT and PolyPhen-2.

Variant ID	Location	Alleles	Source	AA	AA positions	SIFT class	SIFT	PolyPhen-2 Class	PolyPhen-2	Transcript
**rs775900491**	3:122325305	G/T	dbSNP	G/C	5	deleterious	0.02	probably damaging	1.0	ENST00000264474.4
**rs367941220**	3:122325306	G/C/T	dbSNP	G/V	5	deleterious	0.01	probably damaging	1.0	ENST00000264474.4
**rs750925495**	3:122325324	C/A/T	dbSNP	P/H	11	deleterious	0	probably damaging	1.0	ENST00000264474.4
**rs750925495**	3:122325324	C/A/T	dbSNP	P/L	11	deleterious	0.02	probably damaging	0.999	ENST00000264474.4
**rs1183822233**	3:122325329	A/G	dbSNP	T/A	13	deleterious	0	probably damaging	1.0	ENST00000264474.4
**rs1003245034**	3:122325330	C/A	dbSNP	T/N	13	deleterious	0.03	probably damaging	1.0	ENST00000264474.4
**rs1486315855**	3:122337607	T/A	dbSNP	Y/N	43	deleterious	0	probably damaging	1.0	ENST00000264474.4
**rs1262778803**	3:122337608	A/G	dbSNP	Y/C	43	deleterious	0.01	probably damaging	1.0	ENST00000264474.4
**rs1559982616**	3:122337619	G/T	dbSNP	V/F	47	deleterious	0.02	probably damaging	0.987	ENST00000264474.4
**rs745733445**	3:122337622	G/T	dbSNP	V/F	48	deleterious	0	probably damaging	1.0	ENST00000264474.4
**rs1448459675**	3:122337637	T/C	dbSNP	Y/H	53	deleterious	0.02	probably damaging	1.0	ENST00000264474.4
**rs1362356969**	3:122341542	A/T	dbSNP	K/M	91	deleterious	0.02	probably damaging	1.0	ENST00000264474.4
**rs200394711**	3:122341550	G/A/C	dbSNP	E/K	94	deleterious	0.04	probably damaging	0.999	ENST00000264474.4
**rs200394711**	3:122341550	G/A/C	dbSNP	E/Q	94	deleterious	0.04	probably damaging	0.996	ENST00000264474.4

**Table 2:** Analysis of the previously selected deleterious nsSNPs by six tools: PROVEAN, PMut, MutPred2, SNAP2, PHD-SNP, and SNP & GO. nsSNPs that are found to be deleterious by all eight tools are highlighted in yellow.

rsIDs	Variants	PROVEAN		PMut		Mutpred2	SNAP2			SNP & GO		PHD-SNP	
		Score (cutoff = −2.5)	Prediction	Disease Prediction	Prediction score	Score	Score	Expected Accuracy	Predicted Effect	Prediction	RI	Effect	RI
**rs775900491**	G5C	−7.346	Deleterious	TRUE	0.8556	0.71	42	71 %	Effect	Neutral	2	Disease	2
**rs367941220**	G5V	−7.258	Deleterious	TRUE	0.6789	0.775	59	75 %	Effect	Neutral	0	Neutral	2
**rs750925495**	P11H	−7.991	Deleterious	TRUE	0.6686	0.306	34	66 %	Effect	Neutral	5	Neutral	3
**rs750925495**	P11L	−8.958	Deleterious	TRUE	0.5329	0.3	24	63 %	Effect	Neutral	5	Neutral	5
**rs1183822233**	T13A	−4.614	Deleterious	TRUE	0.6686	0.361	33	66 %	Effect	Neutral	7	Disease	0
**rs1003245034**	T13N	−4.722	Deleterious	FALSE	0.4778	0.434	43	71 %	Effect	Neutral	4	Disease	1
**rs1486315855**	Y43N	−8.05	Deleterious	TRUE	0.8046	0.856	83	91 %	Effect	Disease	6	Disease	9
**rs1262778803**	Y43C	−7.783	Deleterious	TRUE	0.7121	0.807	66	80 %	Effect	Disease	6	Disease	7
**rs1559982616**	V47F	−4.301	Deleterious	TRUE	0.6682	0.731	52	75 %	Effect	Disease	0	Disease	7
**rs745733445**	V48F	−4.917	Deleterious	TRUE	0.8293	0.852	76	85 %	Effect	Disease	9	Disease	5
**rs1448459675**	Y53H	−4.428	Deleterious	TRUE	0.561	0.653	69	80 %	Effect	Disease	3	Disease	3
**rs1362356969**	K91M	−4.802	Deleterious	FALSE	0.4121	0.136	5	53 %	Effect	Neutral	8	Neutral	2
**rs200394711**	E94Q	−2.621	Deleterious	TRUE	0.7184	0.191	56	75 %	Effect	Neutral	3	Disease	1
**rs200394711**	E94K	−3.509	Deleterious	TRUE	0.7184	0.634	9	53 %	Effect	Disease	1	Disease	3

### 3.3 Protein stability analysis suggest stability decline in all six deleterious nsSNPs

Six high-risk nsSNPs were then examined using I-Mutant 2.0, MUpro, and the INPS server to assess any alteration that happened in the protein structure. All three servers predicted reduced protein stability for each of the nsSNPs. The free energy change (DGG) values and phenotypic impacts provided by the three servers are shown in Table 3. For all six nsSNPs, DDG changed negatively, ranging from 0.068 to 2.15 kcal/mol, suggesting a certain degree of protein stability decline [70].

**Table 3:** Prediction of nsSNP-mediated alteration in protein stability by I-Mutant 2.0, MUpro, and the INPS server.

Mutation	I-mutant Prediction (Effect)	I-mutant DDG value Prediction (Kcal/mol)	MUpro (Effect)	MUpro (DDG) (Kcal/mol)	I-Mutant Reliability Index (RI)	INPS Stability Changes (DDG) (Kcal/mol)
**Y43N**	Decrease	−1.14	Decrease Stability	−1.12	6	−1.90712
**Y43C**	Decrease	−0.83	Decrease Stability	−0.79	5	−1.27047
**V47F**	Decrease	−1.88	Decrease stability	−0.68	7	−1.86866
**V48F**	Decrease	−0.91	Decrease Stability	−0.65	6	−2.15414
**Y53H**	Decrease	−1.39	Decrease Stability	−1.29	5	−1.26977
**E94K**	Decrease	−0.64	Decrease Stability	−1.37	7	−0.06846

### 3.4 ConSurf analysis reveals conservation scores for five of six CSTA variants

**Fig. 3:**
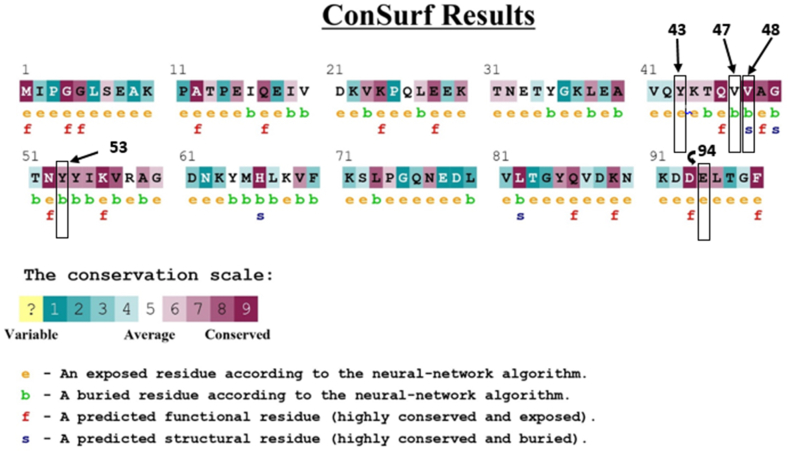
Evolutionary conservation prediction and solvent accessibility analysis of high-risk nsSNPs by ConSurf. SNPs of interest are highlighted in boxes with their respective positions.

The ConSurf server was deployed to measure the evolutionary conservation and solvent accessibility. This browser provides a score for each amino acid position on a scale of 0–9, with a higher score indicating greater conservancy. ConSurf predicted that five of the six high-risk nsSNPs (Y43C, Y43N, V48F, Y53C, and E94K) were conserved. Among them, V48F had the highest conservation score of 9, while Y43C, Y43N, Y53C, and E94K were predicted to be moderately conserved, with scores of 6, 6, 7, and 7, respectively. Only one variant, V47F, was projected as average with a conservation score of 5 and excluded from the downstream analysis. Variations found in conserved areas are considered damaging to the protein, in contrast to those found in non-conserved locations [71]. Out of these nsSNPs, V47F, V48F, and Y53H were found to potentially have structural impacts due to their conservation as buried. Y43C, Y43N, and E94K were found to have functional influence as they were exposed. Fig. 3 presents a summary of ConSurf's predictions for each SNP.

### 3.5 HOPE analysis highlights physicochemical and interaction changes

**Fig. 4:**
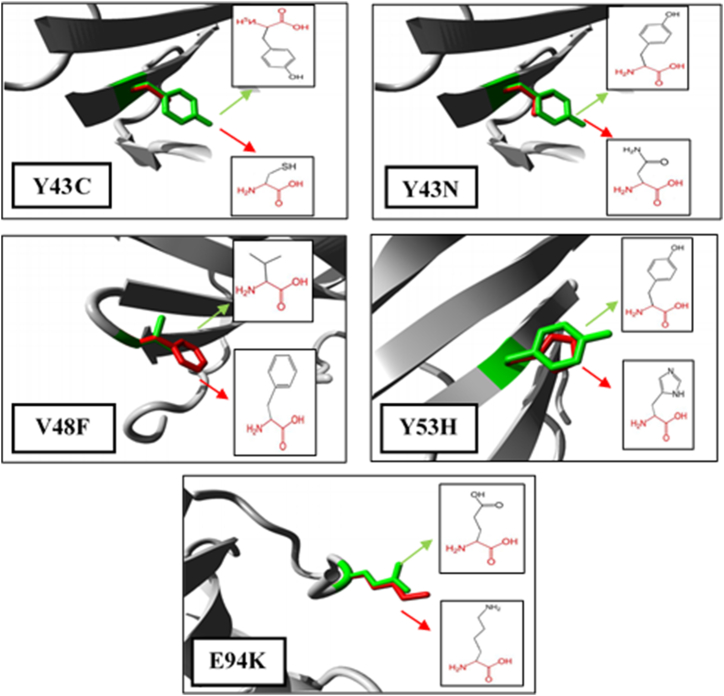
Project HOPE's 3D predictions of the CSTA protein variants. The side chains of the wild-type and mutant residues are colored in green and red respectively.

The Project HOPE server was used to look at how mutations affect different parts of a protein's structure and function, such as its physicochemical properties, hydrophobicity, and interactions between molecules. According to the HOPE server, two mutants (V48F and E94K) were larger than the wild-type residues, whereas three (Y43C, Y43N, and Y53H) were smaller. A Cys (C) residue in the Y43C variant replaced Tyr (Y). This switch could increase hydrophobicity and leave the protein's center with an empty space. Furthermore, this may result in a loss of the hydrogen bond. In the same way, the Y43N variant swapped out the Tyr residue for an Asn (N), which might mean that there were no hydrophobic interactions. Instead of a Val (V), the V48F variant results in a Phe (F) residue. The valine residue at this position is involved in intermolecular contacts, and introducing a bigger residue could disturb these interactions. The Y53H variant produced a His (H) residue instead of Tyr (Y). The Tyr residue at this position formed a hydrogen bond with the Lys (K) residue at position 71, and replacing the native residue with a His (H) may affect the hydrogen bond formation. The E94K variant substituted Lys (K) for the Glu (E). In this instance, the charge has changed from negative to positive. This exchange impacted the salt bridge's development with Lys-44. Diagrams generated by the HOPE server are shown in Fig. 4.

### 3.6 STRING analysis highlights CSTA's strong affinity for Cathepsin B

**Fig. 5:**
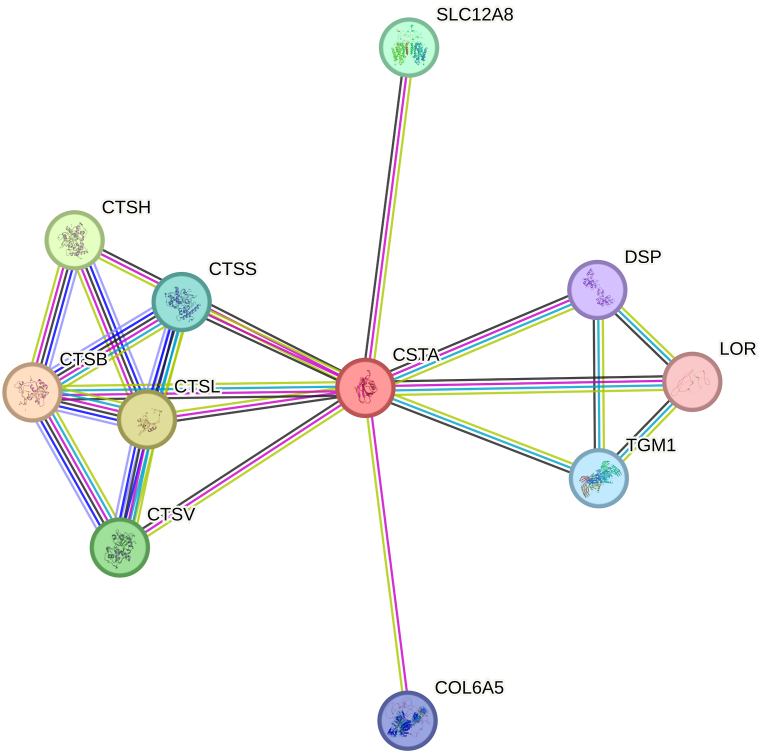
Analysis of the protein–protein interaction network of CSTA. This finding illustrates the interaction partners of the CSTA protein using nodes and edges. The network was created by STRING with a high confidence level of 0.700.

The STRING database displayed ten key interacting molecules with CSTA protein, including Cathepsin B (CTSB), Cathepsin L1 (CTSL), Cathepsin H (CTSH), Cathepsin L2 (CTLV), Solute carrier family 12 member 8 (SLC12A8), Cathepsin S (CTSS), Protein-glutamine gamma-glutamyltransferase K (TGM1), Collagen alpha-5 (VI) chain (COL6A5), Desmoplakin (DSP), and Loricrin (LOR) (Fig. 5; Supplementary File 2). CSTA demonstrated the strongest interaction with cathepsins, specifically with cathepsin B (CTSB), achieving a peak combined score of 0.998. Simultaneously, due to the clinical significance of the CSTA-CTSB interaction in various cancers, CTSB was selected for protein-protein docking and molecular dynamics simulation.

### 3.7 CSTA mutant model generation, refinement, and ramachandran plot analysis ensure structural quality

The five mutant CSTAs were prepared using the SWISS-Model software, utilizing the 3KSE.3.B (X-ray crystallographic structure) as a reference. The sequence identity was 98.98 % for all mutants, and 100 % of queries were covered. Subsequently, the protein models were checked for quality using the QMEAN, GMQE, and QMEANDisCo scores. The QMEAN Z-scores measure protein structure similarity to the native form, with scores close to 0.0 indicating a native structure and scores below −4.0 indicating a low-quality model. Here, scores ranging from −1.22 to −1.45 indicate a high degree of homology with the native structure. The GMQE score in mutant models shows a high level of similarity (about 0.8), which indicates satisfactory model quality. In addition, the QMEANDisCo Global score assesses the quality of models beyond their coverage, and the results have been consistent for all models. Mutant models were energy-minimized and refined using the GalaxyRefine web server. GalaxyRefine generates five refined models for each mutant, selecting the optimal model as the final structure based on criteria like GDT-HA, RMSD, MolProbity, Clash score, Poor rotamers, and Rama preferred amino acids. A higher GDT-HA scoring signifies better overall model quality. The MolProbity score is an important measure of protein quality that combines Clash score, Rotamer, and Ramachandran scores into a single normalized score (Table 4). The root mean square deviation (RMSD) offers valuable information on the average deviation of atoms between the refined and unrefined structure. Ideally, the RMSD should be kept to a minimum. Model refinements were further checked by generating Ramachandran plots using the MolProbity server (version 4.4). Every plot (wild-type/mutant) demonstrated that at least 93 % of their residues were located in the preferred region, classifying them as excellent-quality models [72]. Details of the analysis are given in Table 4.

**Table: 4:** Galaxy Refine and MolProbity scores for the five best-generated CSTA mutants.

GalaxyRefine Server								MolProbity	
CSTA	Model	GDT-HA	RMSD	MolProbity	Clash score	Poor rotamers	Rama favored	Ramachandran Favored (%)	Ramachandran outliers (%)
**Y43C- Mutant**	Raw 3D Model	1	0	2.436	2.6	9.4	85.4	94.79	1.04
	Galaxy refined	0.9617	0.4	1.941	10.2	1.2	94.8		
**Y43N- Mutant**	Raw 3D Model	1	0	2.436	2.6	9.4	85.4	93.75	1.04
	Galaxy refined	0.9592	0.381	1.736	5.1	1.2	93.8		
**V48F- Mutant**	Raw 3D Model	1	0	2.433	2.6	9.4	85.4	95.83	0
	Galaxy refined	0.9668	0.35	1.917	6.3	2.4	95.8		
**Y53H- Mutant**	Raw 3D Model	1	0	1.233	0.7	2.4	96.6	97.75	1.12
	Galaxy refined	0.989	0.283	1.589	8.7	1.2	97.8		
**E94K- Mutant**	Raw 3D Model	1	0	1.114	2.6	1.2	97.9	98.96	1.04
	Galaxy refined	0.9745	0.322	1.414	7.6	0	99		

### 3.8 Binding affinity and Gibbs free energy analysis suggests stability variations in CSTA-CTSB complexes, highlighting E94K as the most unstable

The HDOCK algorithm was utilized to forecast and contrast the various intermolecular interactions between the CSTA and the CTSB. The docking scores for wild-type CSTA and CTSB were −418.22, while all mutant CSTA proteins had similar scores except for Y43N (−395.62) and Y53H (−393.29) variants. The likelihood of an accurate docked pose increases with a lower (more negative) HDOCK docking score, independent of the binding affinity. The docked poses for all protein-protein complexes, with high confidence levels (∼0.99) and negative docking scores, provide a reliable basis for identifying their binding affinities. The stability and binding affinity of the CSTA-CTSB complexes produced by HDOCK were determined by submitting them to the PRODIGY webserver. The Gibbs free energy denoting the stability of the complexes and dissociation constant (K_d_) values, which are inversely proportional to the binding affinity of bound CSTA-CTSB complexes, show changes at physiological temperature (37^o^C). The more negative Gibbs free energy is indicative of the stable receptor-ligand complex. The mutant complexes' Gibbs free energy (ΔG) value remained comparable to the wild-type in the V48F-CTSB complex (−16.2 kcal/mol), increasing for Y43C-CTSB (−17.9 kcal/mol) and Y43N-CTSB (−16.6 kcal/mol), and decreasing for Y53H-CTSB (−15.8 kcal/mol) and E94K-CTSB (−14.9 kcal/mol). Various surface contacts between proteins and non-interacting surfaces may cause these alterations (Table 5). The E94K-CTSB complex exhibited the highest Gibbs free energy (−14.9 kcal/mol) among all the receptor-ligand complexes, indicating the complex is less stable. Again, the binding affinity between the E94K-mutant and CTSB was the lowest (indicated by the highest K_d_ value of 3.40E-11 M), which supports the weaker interactions between them, resulting in a relatively unstable complex compared to the wild-type and other mutants. Table 5 displays the details of the binding scenario.

**Table 5:** Results of the five docked molecules and the control complex (CSTA and CATB). The higher the HDOCK docking score, the higher the possibility of becoming the accurate docked pose. The PRODIGY server calculated the dissociation constant (K_d_) of the docked complex at 37 °C and their type of contacts. The stability and binding affinity of docked proteins are calculated using ΔG (kcal mol^−1^) and K_d_ (M), respectively.

	HDOCK			Prodigy Server									
				Interfacial Contacts (ICs)								Non-Interacting Surfaces (NIS)	
Protein-protein complex	Docking Score	Confidence Score	Ligand RMSD	ΔG (kcal mol^−1^)	K_d_ (M) at 37 °C (Physiological Temperature)	Charged-Charged	Charged-Polar	Charged-Apolar	Polar-Polar	Polar-Apolar	Apolar-Apolar	Charged	Apolar
**Wild-Complex**	−418.22	0.9953	0.56	−16.2	3.80E-12	3	12	27	6	38	77	29.31	32.76
**Y43C-Complex**	−419.56	0.9955	0.53	−17.9	2.30E-13	4	9	22	5	46	73	28.63	32.6
**Y43N-Complex**	−395.62	0.9927	0.36	−16.6	2.00E-12	4	13	23	6	41	69	29.26	32.75
**V48F-Complex**	−405.8	0.994	0.31	−16.2	3.80E-12	3	12	27	6	38	77	29.31	32.76
**Y53H-Complex**	−393.29	0.9924	0.4	−15.8	7.30E-12	2	11	26	6	37	70	28.81	33.05
**E94K-Complex**	−406.11	0.9941	0.62	−14.9	3.40E-11	2	10	22	6	34	71	28.82	32.31

### 3.9 Distinct binding patterns in wild-type and mutant CSTA-CTSB complexes highlight key interaction shifts

**Fig. 6:**
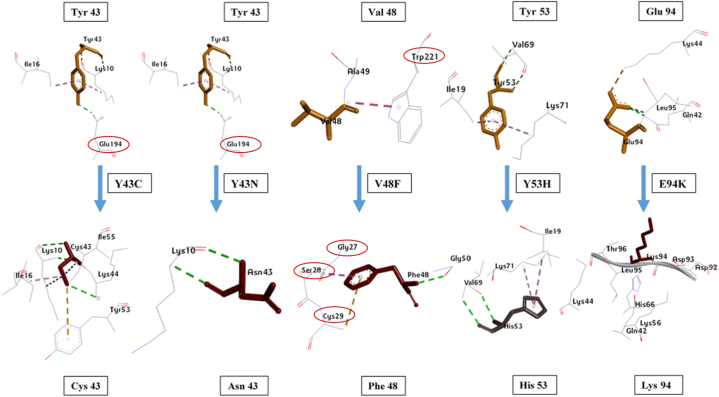
Mutation position-specific amino acid interactions CSTA proteins. Figures show wild-type residues (shown in dark yellow) at the top and mutated residues (shown in maroon) at the bottom. The dotted line displays non-bonded interactions. Interacting CTSB residues are mentioned within red circles.

Identifying the interacting amino acids in a protein-protein docked complex is essential for understanding the conformational changes and binding energy fluctuations. Here, two distinctive but interrelated approaches have been taken. Preliminarily, mutated amino acid residues of interest have been selected to discover how they non-covalently bind with their adjacent amino acids (both intramolecular and intermolecular). At the same time, their binding differences were noted. Wild-type Tyr-43 formed three hydrogen bonds and two hydrophobic interactions (Pi-sigma and -pi alkyl). Here, an intermolecular hydrogen bond was observed with CTSB: Glu-194. On the other hand, mutant Cys-43 formed three hydrogen bonds and three hydrophobic interactions (all intramolecular) with Lys-10, Lys-44, Ile-16, and Ile-55, which are all nearby amino acids. The presence of a sulfur atom in Cys resulted in a unique pi-sulfur bond. The Y43N mutation produced a different result. The aliphatic side chain of Asn-43 significantly reduced the number of non-covalent bonds. Only two intramolecular H-bonds with Lys-10 were visible. Both the Y43C and Y43N mutants lost intermolecular hydrogen bonds with Glu-194 of CTSB. In the case of Val-48, the wild type had only an intermolecular amide-pi bond with CTSB: Trp-221. However, in the V48F mutant, Phe-48 formed bonds both internally and with CTSB. It has an H-bond with Gly-50, a pi-sulfur bond with CTSB: Cys-29, and a stacked amine bond with CTSB: Gly-27 and CTSB: Ser-28. Simultaneously, the V48F mutant lost the Trp-221-mediated hydrophobic interaction. At position 53, the wild-type Tyr formed two hydrogen bonds with the same amino acid, Val-69, and two more hydrophobic interactions were also demonstrated (Ile-19 and Lys-71). The mutant His-53 exhibited a similar pattern despite its different bond lengths. In wild-type CSTA, Glu-94 formed two distinct hydrogen bonds with Gln-42 and Leu-95. Furthermore, the presence of opposing charges caused a strong salt bridge formation with Lys-44 due to the presence of opposing charges. In the E94K mutant, on the other hand, Lys-94 didn't form any non-bonded interactions (Fig. 6).

**Fig. 7:**
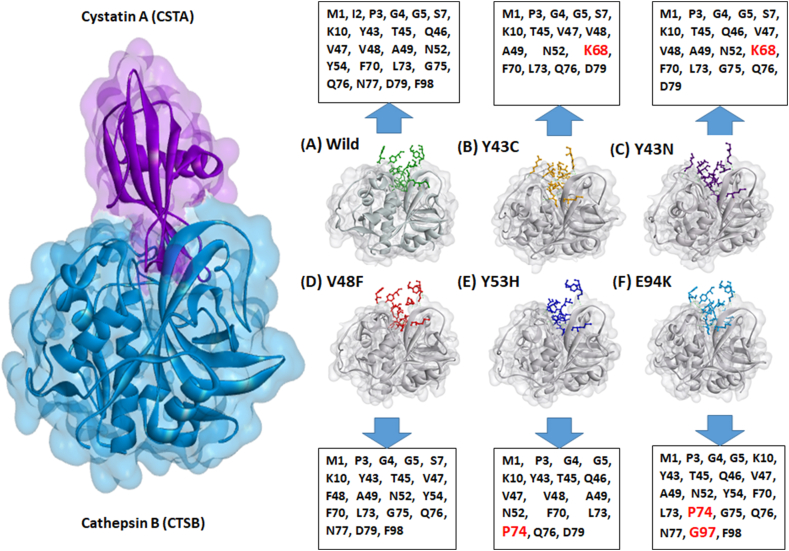
The CSTA (in violet)-CTSB (in blue) interacting complex is shown on the left. The right figure illustrates the Interacting CSTA wild-type and mutants. Gray represents the CTSB, while interacting amino acids of CSTA mutants are colored as follows: wild-type (green), Y43C (yellow), Y43N (purple), V48F (red), Y53H (deep blue), and E94K (sky blue). Amino acid letter codes and respective positions are placed with arrow signs. Highlighted red amino acids are newly adopted in CSTA-CTSB interaction.

Another approach observed the holistic interaction patterns between wild-type and mutant CSTA-CTSB complexes. The binding pocket remained consistent in both; however, the interacting amino acids from CSTA exhibited a degree of divergence. There were several residues that interact with the CSTB active site in both native and mutant CSTA proteins. These included Met-1, Pro-3, Gly-4, Gly-5, Lys-10, Thr-45, Val-47, Ala-49, Asn-52, Phe-70, Lys-73, Gln-76, Asp-79, and Phe-98. However, in mutants, the loss of interacting amino acids was more common than the arrival of new amino acids. The Y43N mutant, for instance, contained only 16 interacting amino acids, whereas the wild-type CSTA had the highest amount of interacting amino acids (22 residues). Notably, Ile-2 was the only interacting amino acid absent in every mutant, whereas Val-48 was only missing in the E94K mutant. The Y43C and Y43N mutants had relatively similar interactions, with Lys-68 being a newly arrived interacting amino acid in both mutants. It's intriguing to note that the mutant residue at position 43 was unable to participate in this interaction. In the Y53H mutant, Pro-74 was the only interacting residue that gained a function, whereas the E94K mutation gains two new interacting residues, Pro-74 and Gly-95. Fig. 7 provides details of the interaction.

### 3.10 Molecular dynamics analysis reveals conformational changes and stability variations in wild-type and mutant CSTA-CTSB complexes

**Fig. 8:**
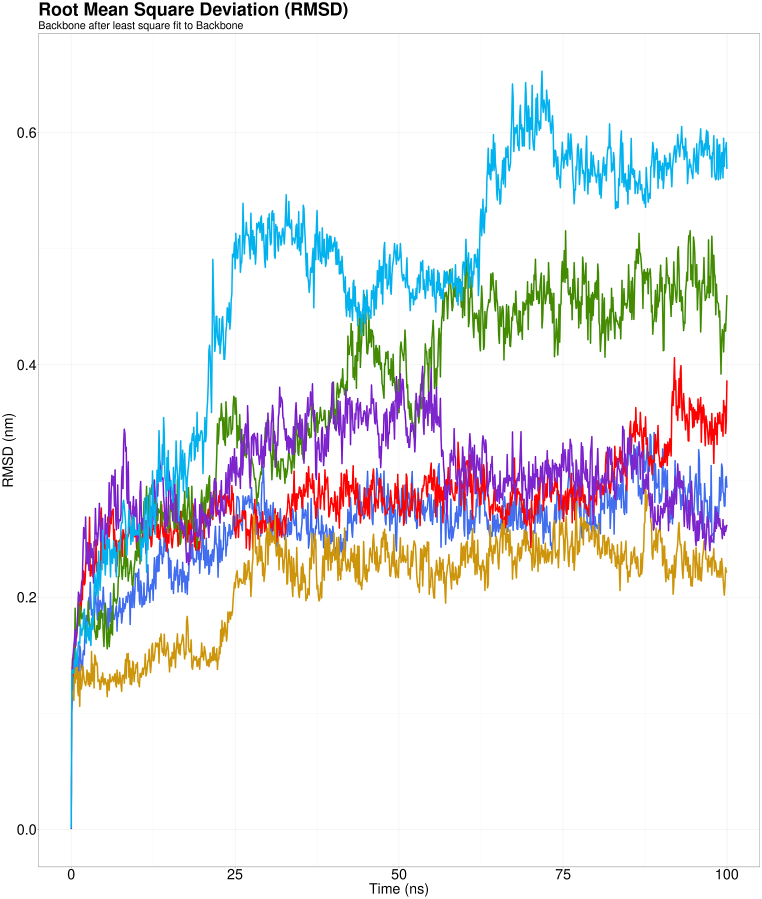
RMSD analysis of wild-type CSTA-CTSB complex (shown in green), Y53H-CTSB complex (shown in deep blue), E94K-CTSB complex (shown in sky blue), V48F-CTSB complex (shown in red), Y43C-CTSB complex (shown in yellow), and Y43N-CTSB complex (shown in purple).

The dynamic behavior of the native and mutant complexes was investigated in response to the results of the docking analysis. Several parameters, including RMSD, RMSF, Rg, and SASA, were considered between the native and mutant CSTA-CTSB complexes. Firstly, RMSD calculations were carried out to assess the stability of the systems. The stability of a complex structure is indicated by a lower RMSD value [73]. As a result of protein's conformational changes brought on by single nucleotide variations and ligand binding, variations in RMSD value were evident. The wild-type protein-ligand complex in Fig. 8 (shown in green) exhibited a steady increase in RMSD. The E94K-CTSB (shown in sky blue) complex showed markedly higher RMSD compared to the wild-type, suggesting significant conformational change and subsequent instability of the complex. The Y43N-CTSB (shown in purple) complex maintained a similar RMSD profile compared to the wild-type till around 50 ns. After that, it declined sharply. V48F-CTSB (shown in red) and Y53H-CTSB (shown in deep blue) complexes possessed relatively low RMSD values, while Y43C-CTSB (shown in yellow) complexes had the lowest RMSD value. The variation in RMSD values significantly affected the understanding of protein-ligand interactions, their stability, and the potential functional changes they may induce.

**Fig. 9:**
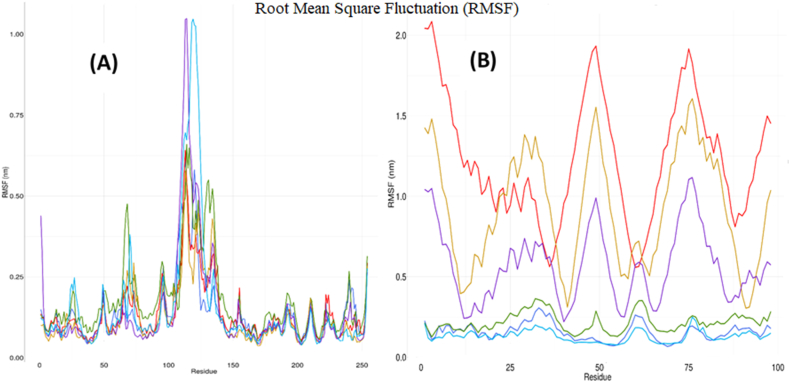
RMSF analysis of CTSB and CSTA has been shown. Fig. (a) shows the flexibility of all amino acids in CTSB when interacting with both wild and mutant CSTA. In Fig. (b), patterns of deviations in CSTA are mentioned. In each instance, the wild proteins are depicted in green, the interacting CTSB and Y53H are depicted in deep blue, the interacting CTSB and E94K are depicted in sky blue, the interacting CTSB and V48F are depicted in red, the interacting CTSB and Y43C are depicted in yellow, and lastly, the interacting CTSB and Y43N are depicted in purple.

The regional flexibility of specific amino acid positions was evaluated by RMSF, providing a detailed understanding of protein dynamics. Lower RMSF values align with stiff structures, whereas higher RMSF values align with protein flexibility [73]. The wild-type CTSB's regional adaptability was depicted in Fig. 9 (a), which indicated that the area between the 100th and 150th residues detected the most variation. When interacting with Y43N and E94K mutants, CTSB exhibited the most variable pattern. The regional flexibility of the wild-type and mutant CSTAs as a result of binding with the CTSB is depicted in Fig. 9 (b). It has been found that the V48F, Y43C, and Y53N mutants demonstrated the highest flexibility, whereas the E94K mutant displayed the lowest.

**Fig. 10:**
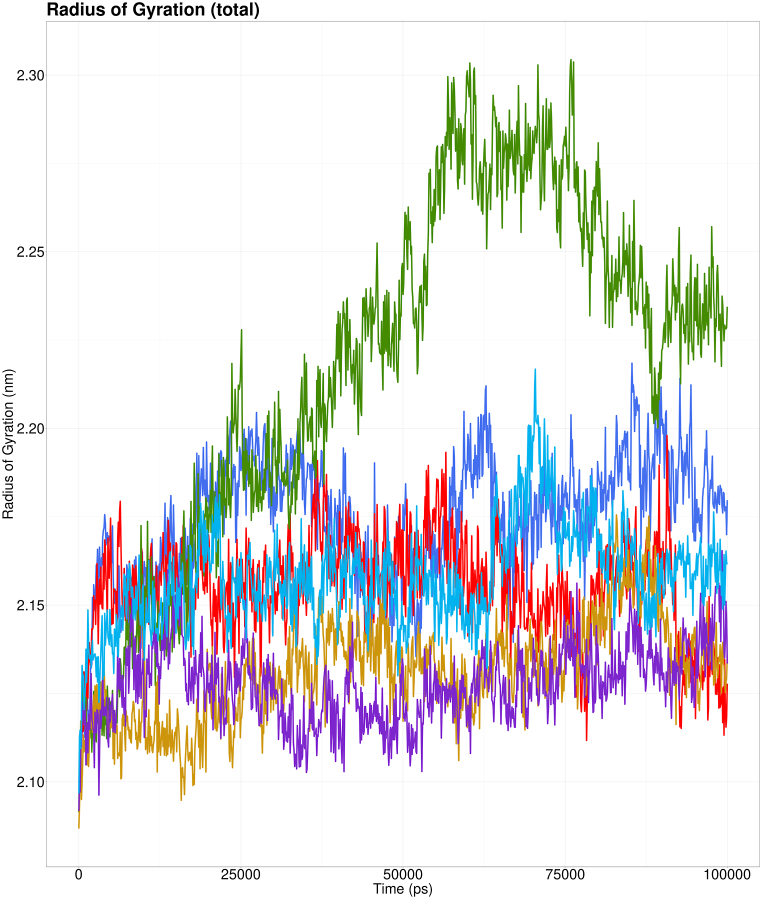
Rg analysis of wild-type CSTA-CTSB complex (shown in green), Y53H- CTSB complex (shown in deep blue), E94K- CTSB complex (shown in sky blue), V48F- CTSB complex (shown in red), Y43C- CTSB complex (shown in yellow), and Y43N- CTSB complex (shown in purple).

When Rg is reasonably constant, a protein folds steadily, whereas when it fluctuates, it indicates that the protein is unfolding [73]. The Rg analyses of the wild-type CSTA-CTSB complex (shown in green) revealed that it has the greatest and most fluctuating Rg, indicating the unfolding nature and a low level of protein compactness. In contrast, the remaining complexes have relatively constant Rg values and hence showed a more compact structure, indicating a different pattern of ligand interaction. These Rg values are crucial in understanding the folding and unfolding process of the protein (Fig. 10).

**Fig. 11:**
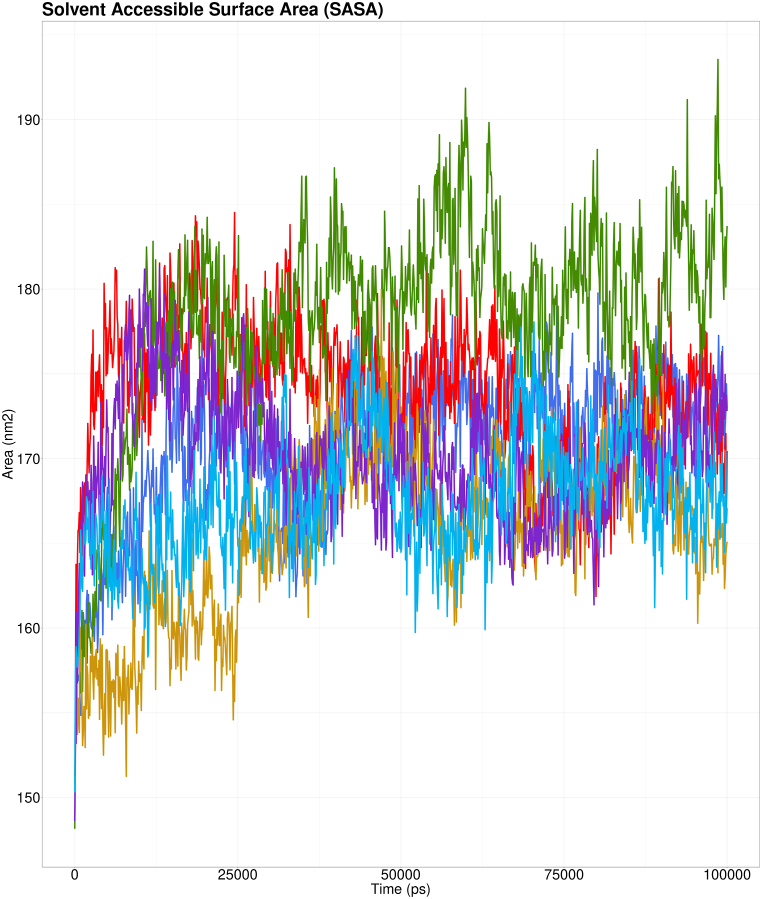
SASA analysis of wild-type CSTA-CTSB complex (shown in green), Y53H-CTSB complex (shown in deep blue), E94K-CTSB complex (shown in sky blue), V48F-CTSB complex (shown in red), Y43C-CTSB complex (shown in yellow), and Y43N-CTSN complex (shown in purple).

In molecular dynamics simulations, SASA is used to predict the solvent exposure of proteins' hydrophobic cores. Higher SASA values imply that much of the protein is exposed to water, while lower values imply that much of the protein is buried inside the hydrophobic core [73]. SASA values indicated that all CSTA mutants-CTSB complexes were less exposed to solvents than the wild-type CSTA-CTSB complex (shown in green). These mutations are likely to cause alteration in the functioning of the CSTA protein at different degrees (Fig. 11).

## 4 Discussion

The Cystatin A *(CSTA)* gene encodes for a class of inhibitors that block the proteolysis of cytoplasmic and cytoskeletal proteins by cathepsins. Enzyme kinetics research has identified the altered affinity between CSTA and cathepsin B by creating mutations at various places within the complex [74]. G4A and G4S mutations in CSTA can cause decreased affinity for CTSB by ∼5000 and ∼25000 folds, respectively, while increasing their net dissociation constant K_d_ by ∼40 and ∼640 folds [74]. A separate study found that L73G and P74G mutants reduced cathepsin B affinity by 4000 and 10-fold, respectively, while Q76G and N77G mutants did not show significant effects [75]. Though these studies provide valuable insights, they only focus on a few important structural positions. Concurrently, a comprehensive, methodical investigation to elucidate the structural and functional impacts of all existing missense SNPs is still lacking.

The occurrence of deleterious nsSNPs in cancer-associated genes has caught attention in recent years, leading to a growing need for *in silico* analysis of SNPs in large datasets [76,77]. For a comprehensive understanding of the impact of SNPs, it is advisable to utilize four or five algorithmic tools [78]. However, this study identified six deleterious nsSNPs (“Y43C,” “Y43N,” “V47F,” “V48F,” “Y53H,” and “E94K”) through an integrated computational approach, involving eight tools to boost confidence and get a full picture of the pathogenic SNPs. The level of accuracy, prediction approaches, and algorithms of these tools vary, and their web servers are regularly updated, resulting in potential differences in prediction results [79].

Essentially, structural instability leads to the breakdown, misfolding, and aggregation of proteins, ultimately resulting in dysfunction [80,81]. Protein stability analysis showed reduced values for all selected variants, indicating destabilization of protein structure. However, all positional mutations are not equally harmful in terms of evolutionary perspective [82]. ConSurf profiling revealed five of the six destabilizing mutations occur at conserved residues, emphasizing their potential to affect protein function. Destabilizing nature within the evolutionary conserved positions prompted further investigation into their altered structure. Here, Project HOPE revealed notable disruptions in physicochemical properties and CSTA interaction networks, including the loss of hydrogen bonds, modified hydrophobic core interactions, and altered molecular packing. Moreover, E94K mutation replaces a negatively charged glutamic acid with a positively charged lysine, leading to the disruption of an essential salt bridge with Lys-44. This modification likely reduces structural integrity and decreases functional efficiency, highlighting its potential to significantly compromise CSTA's regulatory role. These alterations revealed a possible effector mechanism for missense variants, but they are not absolute and occasionally deceiving [83,84].

Since structural alterations are not limited to intramolecular interactions, the network of protein molecules has a significant impact on understanding biological processes. CSTA was seen to interact with the following five cathepsins: cathepsin B, H, L, S, and V in its default configuration. Among them, the top-scoring cathepsin B was chosen to compare the inhibition capability of CSTA mutants with wild-type protein. Protein-protein docking was conducted with two primary objectives: to ascertain the optimal conformation of CSTA in relation to CTSB and to evaluate the binding affinities linked to that orientation. Both factors are taken into account, as the HDOCK tool focuses on identifying the optimal complex, while PRODIGY assesses the affinities in the specific orientation. The HDOCK docking scores varied from −393.29 to −419.56, with confidence scores exceeding 0.99 in all complexes, indicating the accurate binding pose for both the wild-type and mutant CSTA. The PRODIGY server processed the docked complexes to ascertain their optimal binding affinity and to evaluate any alterations in binding due to mutations. The variations in binding affinity between CSTA mutants and CTSB, in comparison to the native protein, were inconsistent. The E94K-CTSB complex demonstrated the lowest binding affinity, reflected in its highest Kd value of 3.40E-11 M. This indicated that their interactions are less robust, leading to a comparatively unstable complex in relation to the wild-type and other mutants. The complex also exhibited a Gibbs free energy of −14.9 kcal/mol, suggesting its instability once more. These results demonstrate that the E94K mutant is unable to effectively inhibit CTSB protease activity, resulting in cellular imbalance and potential disease progression.

The relationship between the number of interacting amino acid residues and binding affinity is not straightforward; an increase in residues does not necessarily correlate with higher binding affinity, and vice versa. Despite losing an H-bond with CTSB: Glu-194 (Fig. 6), the Y43C and Y43N variants displayed higher binding affinities and fewer CSTA residues involved in complex formation (Fig. 7). Among the polymorphic sites, Y43 and V48 participated in the wild-type CSTA-CTSB interaction. Both the Y43C and Y43N variants lost the Y43 interaction and gained an additional interacting K68 residue, which might account for favorable complex formation with higher binding affinities. Instead, V48F and E94K had almost the same number of interacting amino acid residues as the wild-type complex (Fig. 7), but their binding affinities were not the same. In the V48F variant, the loss (CTSB: Trp-221) and addition (CTSB: Gly-27, Ser-28, and Cys-29) of bond with CTSB have no effect on their affinity (Fig. 6). Other notable interacting residues of CSTA, such as S7, Y43, and F48, remained similar to the wild-type complex, leading to the same binding affinity, whereas E94K showed lower affinity. The interactions of P74 and G97 with CTSB in E94K may not match the bonding strength of the native interactions that were lost (e.g., S7, V48). Additionally, the substitution of Glu-94 with Lys (Fig. 6) may result in the loss of hydrogen bonds and the formation of a distinct salt bridge, potentially inducing a conformational change in the CSTA structure. This alteration could decrease the binding affinity for CTSB and hinder the formation of a stable CSTA-CTSB complex. This observation parallels the findings from Project HOPE concerning salt bridge formation in the E94K mutant.

While molecular docking helps produce preliminary binding hypotheses, molecular dynamics simulations provide a deeper understanding of these interactions with high atomic precision [85]. It has demonstrated utility in deciphering the modes of action of proteins and other biomolecules, as well as in identifying the structural basis of disease [86]. The physical basis of the structure-to-function link of wild-type and mutant proteins was seen by evaluating four parameters using MD simulation (100 ns): RMSD, RMSF, Rg, and SASA. The Y43C, V48F, and Y53N mutants were more flexible in all amino acid positions, but the complex remained stable in RMSD measures. However, the E94K-CTSB complex, unlike others, exhibited a higher RMSD value (>5 Å), suggesting a significant deviation in the stability. This finding aligns with earlier molecular docking data, indicating that this complex exhibited the lowest binding affinity (−14.9 kJ/mol). RMSF gives a quantitative score for individual amino acid fluctuations to understand the flexibility pattern inside a protein [73]. CTSB showed increased flexibility between the 100th-150th residues across all complexes. The E94K mutant demonstrated minimal fluctuations in RMSF; however, the docked protein complex displayed increased RMSD. The findings indicate that the E94K mutant lacks the necessary flexibility for optimal binding with CTSB. Simultaneously, all CSTA variants exhibited comparable Rg and SASA patterns, displaying notable deviations from the wild type; however, the results for the E94K mutant align with the interpretations of both the RMSD and RMSF datasets. The SASA analysis of the E94K-CTSB complex indicated a potential reduction in solvent exposure, which may influence the accessibility of critical cystatin-cathepsin interaction sites. It is also demonstrated by a consistently lower Rg during the entire simulation, indicating changes in compactness and a reduction in protein volume. The findings indicate that wild-type CSTA, upon high-affinity binding, unfolds and enhances the surface accessibility of CTSB, consequently inhibiting its conventional protease activity. All variants, with the exception of E94K, exhibited notable structural diversity; however, their impact on stability and interaction dynamics was comparatively minimal. The E94K variation appears to be less effective in inhibiting CTSB due to its loose binding and the formation of a less stable complex. This enables the CTSB to preserve its compact structure, thereby sustaining uncontrolled proteasomal activity and resulting in abnormal physiological conditions. All the *in silico* tools uniformly revealed the E94K variant affecting the CSTA structure and function, while results of other variants fluctuated, hindering to draw a comprehensive scenario of their deleterious effects.

Two SNPs reported as deleterious in this study, p.Y43C (Tyr43Cys) and p.E94K (Glu94Lys), have been documented in the COSMIC database as present in cancer patients [87]. The presence of the CSTA variants in cancer patients provides additional support to these findings of the Y43C and E94K polymorphisms having detrimental effects on the protein function. The Y43C mutation has been identified in liver neoplasm, while the E94K mutation has been observed in two male patients diagnosed with colorectal adenocarcinoma [87]. From the positional aspects, the Y43C mutation resides in the core beta-sheet structure, which is associated with structural integrity, has been identified, and its alteration may induce rapid protein turnover. Conversely, the E94 mutation is present in the c-terminal loop, and altering it could potentially decrease the flexibility required for binding with cathepsins.

Naturally, CSTA is recognized as a cathepsin B (CTSB) inhibitor maintaining a balanced proteolytic activity under normal physiological conditions through CSTA-CTSB complex formation. The failure of CSTA to effectively inhibit CTSB, caused by genetic alterations including E94K as evidenced in several bioinformatics-based prediction tools, molecular docking, and dynamic simulation in this study, may lead to CTSB dysregulation which can subsequently result in significant ECM degradation, promoting tumor cell migration, and facilitating metastasis [88]. Upregulated CTSB can proteolytically degrade the ECM components including collagen I, collagen IV, fibronectin, and laminin, to drive tumor cell invasion [89]. Inability of mutated CSTA to bind and regulate CTSB activity can also promote angiogenesis by CTSB degrading matrix metalloproteinase inhibitors (TIMPs), which in turn activate MMP-9 and remodel ECM to release sequestered pro-angiogenic factors, including vascular endothelial growth factor (VEGF) and transforming growth factor (TGF) [89]. CTSB also displays a dual role in apoptosis, capable of promoting cell death under regulated circumstances or enhancing survival by initiating autophagy in cancer cells [90]. Altered cystatin activity of the E94K variant may disrupt this equilibrium, allowing tumor cells to escape apoptosis and increase their treatment resistance.

As multiple CSTA variants of clinical significance are emerging, a rapid variant assessment may be necessary. However, this study has several limitations. Phenotypes, for example, cannot be fully predicted simply by genotype because some disease-causing alleles do not show up as a phenotype because of varying penetrance. Furthermore, information regarding the phenotype of complex genetic characteristics, such as cancer, is unlikely to be obtained from a single nsSNP. Additionally, different organs and tissue types throughout the body may have varied expressions and effects from a single mutation [91]. To identify the nsSNPs of CSTA associated with malignancy, it is essential to implement effective experimental procedures in future studies.

## 5 Conclusion

This is the first comprehensive computational analysis of missense SNPs in the CSTA gene, revealing their structural and functional features affecting CTSB inhibition. Five nsSNPs (Y43C, Y43N, V48F, Y53H, and E94K) have been identified as potentially harmful due to their presence in the conserved region and probable impact on protein stability. The E94K mutation significantly affects CTSB binding affinity and the CSTA-CTSB complex, potentially disrupting its regulation and enhancing its proteolytic activity. This could lead to increased tumor invasion and metastasis during cancer progression, as the mutant CSTA is less effective in inhibiting its enzymatic functions. However, while computational predictions provide valuable directions, comprehensive *in vitro* and population-based studies are essential to validate the real-time effects.

## CRediT authorship contribution statement

**Shafaat Hossain:** Writing – original draft, Methodology, Formal analysis, Data curation. **Omar Hamza Bin Manjur:** Writing – original draft, Visualization, Software, Methodology, Investigation, Formal analysis, Data curation, Conceptualization. **Mst Sharmin Sultana Shimu:** Visualization, Methodology, Formal analysis. **Tamanna Sultana:** Visualization, Validation, Methodology. **Mustafizur Rahman Naim:** Writing – review & editing, Visualization, Validation. **Shahariar Siddique:** Writing – review & editing, Visualization. **Abdullah Al Mamun:** Validation, Resources. **Md Miftaur Rahman:** Methodology. **Md Abu Saleh:** Writing – review & editing, Formal analysis. **Md Rakibul Hasan:** Writing – original draft, Resources. **Tania Rahman:** Writing – review & editing, Writing – original draft, Supervision, Project administration, Investigation, Conceptualization.

## Data availability statement

The data can be obtained by submitting a formal request to the corresponding author.

## Funding

This study did not receive any specific grant funding from public, private, or non-profit organizations.

## Declaration of competing interest

The authors declare that they have no known competing financial interests or personal relationships that could have appeared to influence the work reported in this paper.
